# Pulmonary Alveolar Stem Cell Senescence, Apoptosis, and Differentiation by p53-Dependent and -Independent Mechanisms in Telomerase-Deficient Mice

**DOI:** 10.3390/cells10112892

**Published:** 2021-10-26

**Authors:** Kexiong Zhang, Lihui Wang, Xiaojing Hong, Hao Chen, Yao Shi, Yingying Liu, Jun Liu, Jun-Ping Liu

**Affiliations:** 1 Institute of Ageing Research, Hangzhou Normal University School of Basic Medical Sciences, Hangzhou 311121, China; wanglihui100@163.com (L.W.); xiaojing.hong@hznu.edu.cn (X.H.); chenhao502@126.com (H.C.); shiyaowow@163.com (Y.S.); 11111010067@fudan.edu.cn (Y.L.); junliu262@hznu.edu.cn (J.L.); 2 Hudson Institute of Medical Research and Monash University Department of Molecular and Translational Science, Clayton, VIC 3168, Australia; 3 Department of Immunology and Pathology, Monash University Faculty of Medicine, Prahran, VIC 3181, Australia

**Keywords:** telomerase RNA component, telomere shortening, lung alveolar type 2 cells, senescence, apoptosis, differentiation, p53

## Abstract

Pulmonary premature ageing and fibrogenesis as in idiopathic pulmonary fibrosis (IPF) occur with the DNA damage response in lungs deficient of telomerase. The molecular mechanism mediating pulmonary alveolar cell fates remains to be investigated. The present study shows that naturally occurring ageing is associated with the DNA damage response (DDR) and activation of the p53 signalling pathway. Telomerase deficiency induced by telomerase RNA component (TERC) knockout (KO) accelerates not only replicative senescence but also altered differentiation and apoptosis of the pulmonary alveolar stem cells (AEC2) in association with increased innate immune natural killer (NK) cells in *TERC* KO mice. *TERC* KO results in increased senescence-associated heterochromatin foci (SAHF) marker HP1γ, p21, p16, and apoptosis-associated cleaved caspase-3 in AEC2. However, additional deficiency of the tumour suppressor p53 in the *Trp53*^−/−^ allele of the late generation of *TERC* KO mice attenuates the increased senescent and apoptotic markers significantly. Moreover, p53 deficiency has no significant effect on the increased gene expression of *T1α* (a marker of terminal differentiated AEC1) in AEC2 of the late generation of *TERC* KO mice. These findings demonstrate that, in natural ageing or premature ageing accelerated by telomere shortening, pulmonary senescence and IPF develop with alveolar stem cell p53-dependent premature replicative senescence, apoptosis, and p53-independent differentiation, resulting in pulmonary senescence-associated low-grade inflammation (SALI). Our studies indicate a natural ageing-associated molecular mechanism of telomerase deficiency-induced telomere DDR and SALI in pulmonary ageing and IPF.

## 1. Introduction

Mammalian lung tissue plays an important role in O_2_ and CO_2_ gas exchange through numerous alveoli. The alveolus has a semi-circular shape, serving as the basic physiological unit in lung parenchyma. Alveoli are comprised of alveolar epithelia and interstitial space [1]. Two major cell types of alveolar epithelia are alveolar epithelial type 1 (AEC1) cells and alveolar epithelial type 2 (AEC2) cells. As smaller and rounder cells than AEC1 cells, AEC2 cells function to secrete surfactant proteins and lipids to maintain alveolar surface tension and differentiate as stem cells in alveolar epithelia in lung tissue [2,3,4]. When AEC1 cells are injured, AEC2 cells directly differentiate into AEC1 cells to offset epithelial loss [2,5,6]. Importantly, recent studies have revealed that AEC2 cells act as adult stem cells in the lungs [2,4,5,6,7].

Lung function is thought to begin declining at approximately 25 years of age in humans [8]. Consequently, the incidence of age-dependent diseases, such as idiopathic pulmonary fibrosis (IPF) and chronic obstructive pulmonary disease (COPD), are significantly increased in adults older than 45 years of age [9]. The aetiologies of the diseases remain unclear. From genetic studies, >40% of familial IPF patients and 20% of sporadic IPF patients showed significant telomere shortening [10,11,12,13,14]. In COPD patients, telomere lengths were found to be significantly shorter than those in healthy controls [15,16,17]. However, the connection between telomere shortening and the genesis of these two disorders is largely unknown.

Telomere length is shortened with each cell division. In contrast to this, telomere length is maintained by telomerase, which contains a telomerase RNA component (TERC) and telomerase reverse transcriptase (TERT) [18]. TERC functions as a template to add the telomere repeat sequence to the end of telomeres under the catalysis of TERT [18,19,20]. Human TERC was reported to encode a 121 amino acid protein termed hTERP with a potential role against apoptosis [21]. The *TERC* knockout mouse model shows significantly shortened telomeres in late generations [18,19] and has been used to investigate the physiological effects of telomere shortening in stem and progenitor cells, including the hematopoietic and pulmonary systems [20]. Currently, telomere shortening triggers stem cell senescence and apoptosis, impairing tissue repair and regeneration [4,22,23]. 

Genetic and molecular investigations indicate that telomere shortening induced by TERT disruption or telomere dysfunction without telomere shortening in AEC2 cells induces pulmonary fibrosis in mice [24]. Recently, we demonstrated that severe stress such as bacterial toxin bleomycin or radiation trigger pulmonary premature ageing and fibrosis by telomere uncapping [4]. Through stress-related protein kinase GSK3β phosphorylation and subsequent ubiquitin E3 ligase FBW7 ubiquitination of telomere protein TPP1, the telomere DNA damage response (DDR) mediates reversible AEC2 replicative senescence and pulmonary fibrosis [4,25]. In addition, we also demonstrated that telomerase deficiency due to either *TERC* or telomerase catalytic subunit *TERT* knockout induces pulmonary fibrosis with AEC2 senescence and senescence-associated low-grade inflammation (SALI) [20,26]. However, the mechanisms whereby telomere DDR causes SALI and fibrosis remain to be investigated [26,27].

The present study demonstrates that naturally aged mice showed DDR with an activated p53 pathway in association with pulmonary ageing and cellular senescence. Telomerase inactivation by *TERC* gene deletion accelerated not only senescence but also apoptosis and differentiation in AEC2 cells by p53-dependent and -independent mechanisms. Furthermore, we found that increased innate immune NK cells were involved in the pathophysiology pulmonary senescence induced by telomerase deficiency and telomere DDR in the mouse lungs.

## 2. Materials and Methods 

### 2.1. Mice

Eighteen- and thirty-month-old wild-type mice were used in this study. *Terc*^+/−^ mice [19,28] with a C57BL/6J background were inter-crossed to generate *Terc*^+/+^ mice and first-generation G1 *Terc*^−/−^ mice. These G1 *Terc*^−/−^ mice were crossed successively to produce second-generation G2 *Terc*^−/−^ mice and third-generation G3 *Terc*^−/−^ mice to acquire mice with significantly shorter telomeres. *Terc*^+/−^ mice were crossed with *p53*^+/−^ [29] mice to produce *Terc*^+/−^*p53*^+/−^ mice. These mice were inter-crossed to generate G1 *Terc*^−/−^*p53*^+/−^. G2 *Terc*^−/−^ *p53*^−/−^ mice are the offspring from crossing G1 *Terc*^−/−^ *p53*^+/−^ with G1 *Terc*^−/−^*p53*^+/−^. The wild-type (WT) control mice were C57BL/6J. The mice were raised under standard conditions in the animal centre of Hangzhou Normal University. The mice that were 2–5 months old were used for all experiments conducted in this work. The Animal Ethics Committee of Hangzhou Normal University approved all surgical procedures (ethical approval code 2016036, 2018012 and 2021-1059).

### 2.2. Respiration Function Assay

The mice were placed into a head-out single chamber plethysmograph for 3–5 min. Data acquisition was performed by the eSpira Forced Manoeuvers System. 

### 2.3. Fibrosis-Associated Masson’s Staining, Collagen Volume Fraction

The lung fibrosis was evaluated by Masson’s trichrome staining. Mice lung tissues were fixed and paraffin embedded. The paraffin sections were cut into 4 μm and stained with Masson trichrome (Sigma) according to the manufacturer’s instructions. The collagen volume fraction was calculated by ImageJ software according to the formula collagen area/total area ∗ 100%.

### 2.4. In Situ Senescence-Associated (SA)-β-Gal Staining in Lung Tissues

Fresh mouse lung tissues were immersed in a fixation solution containing 2% formaldehyde and 0.2% glutaraldehyde in phosphate buffered saline (PBS) for 45 min, then transferred to 30% sucrose, and fixed overnight. The tissues were cut into 6 μm thick cryosections, which were then fixed and incubated overnight at 37 °C with the staining mixture supplied in the Senescence Cells Histochemical Staining Kit (Sigma-Aldrich, St. Louis, MO, USA) before being counterstained with Nuclear Fast Red. Finally, the slides were scanned and photographed with a Pannoramic MIDI II digital slide scanner (3DHISTECH, Budapest, Hungary).

### 2.5. Double Immunofluorescence Staining Analysis

In brief, the 6 μm thick lung cryosections were fixed using 1% paraformaldehyde and then permeabilised with 0.5% SDS. The following primary antibodies were used: anti-Surfactant Protein C (SPC), anti-p21, anti-p16 (Santa Cruz Biotechnology, Inc., Dallas, TX, USA), anti-HP1γ (heterochromatin protein 1γ) (Cell Signaling Technology, Danvers, MA, USA), and anti-T1α (Abcam, Cambridge, UK). The secondary antibodies were Alexa Fluor 488-conjugated donkey anti-goat IgG, Alexa Fluor 555-conjugated donkey anti-rabbit IgG (Molecular Probes, Eugene, OR, USA), and Alexa Fluor 488-conjugated goat anti-Syrian hamster (Jackson ImmunoResearch Laboratories, Inc., West Grove, PA, USA). The cell nuclei were counterstained with 4′6-diamidino-2-phenylindole dihydrochloride. Images were acquired with an Axio imager M2 fluorescence microscope (Carl Zeiss AG, Oberkochen, Germany). The stained rates of p21, p16, and Hp1γ stain in AEC2 cells were calculated using the number of double-stained cells from SPC and one of either p21, p16, or Hp1γ, dividing the number of stained cells by SPC. The T1α stained rates were calculated by dividing the number of T1α stained cells by the total cells labelled with 4′6-diamidino-2-phenylindole dihydrochloride.

### 2.6. Isolation of AEC2 Cells from Lung Tissues by Fluorescence-Activated Cell Sorting (FACS)

AEC2 cells were isolated from lung tissues using a FACS sorter, as previously described (Messier et al., 2012; Fujino et al., 2012). In brief, lung tissues were digested with Dispase II, collagenase (Roche Diagnostics, Indianapolis, IN, USA) and DNase I (Sigma-Aldrich, St. Louis, MO, USA) in order to acquire a single-cell suspension. After removing the large cells and debris with a 100 μm filter, the remaining cells were labelled with PE-CY5.5-conjugated anti-CD45 (eBioscience, San Diego, CA, USA), APC-conjugated EpCAM (BioLegend, San Diego, CA, USA), and PE-conjugated T1α (Biolegend, San Diego, CA, USA) and then run through a BD influx flow cytometer (BD Biosciences, San Jose, CA, USA) in order to harvest AEC2 cell subpopulations with a >90% CD45^−^EpCAM^+^ or CD45^−^EpCAM^+^ T1α^−^ profile.

### 2.7. Real-Time Quantitative PCR

The RNA levels were assayed in both lung tissue and FACS-isolated AEC2 cells by quantitative PCR (qPCR) using the gene-specific primer sequences shown in Supplementary Table S1. Fresh lung tissues were perfused with PBS in order to remove blood cell contamination, followed by snap-freezing and storage at −80 °C. Total RNA from lung tissue was extracted with TRIzol reagent (Life Technologies, Carlsbad, CA, USA) according to the manufacturer’s instructions. Total RNA from AEC2 cells was extracted with an RNeasy Micro Kit (Qiagen, Valencia, CA, USA). Equal amounts of total RNA from lung or AEC2 cells was reverse-transcribed into cDNA using the PrimeScript RT Reagent Kit (Takara Bio (Dalian), Dalian, China). Gene expression was quantified by qPCR using iQ SYBR Green Supermix (Bio-Rad Laboratories, Hercules, CA, USA) with the Bio-Rad CFX96 real-time PCR system. The cycle threshold value of each target gene was normalised to the housekeeping β-actin content of each cDNA and either the 2(−∆Ct) or 2(−∆∆Ct) method was used to calculate and graph the relative mRNA level (ratio to β-actin) or relative expression (as a fold-activation value) [30]. The primer sequences were listed in Supplementary Table S1.

### 2.8. Statistical Analysis

The results were expressed as the mean ± standard error and were based on the indicated number of lung samples per group. For lung staining, four random images from different regions on a randomly selected slide at 20- or 40-fold amplification for each mouse were captured, and the number of stained cells was counted for each image. The average number of stained cells from these four images represented the value of the lung from which the section was cut. The statistical significance of differences was assessed by a two-tailed Student’s *t*-test when two groups were compared or by one-way ANOVA analysis with Bonferroni correction when multiple comparisons were conducted using GraphPad Prism software, with statistical significance indicated by * *p* < 0.05, ** *p* < 0.01, and *** *p* < 0.001.

## 3. Results

### 3.1. Age-Related Activation of the p53 Signalling Pathway in Pulmonary Fibrosis in Mice 

**Figure 1:**
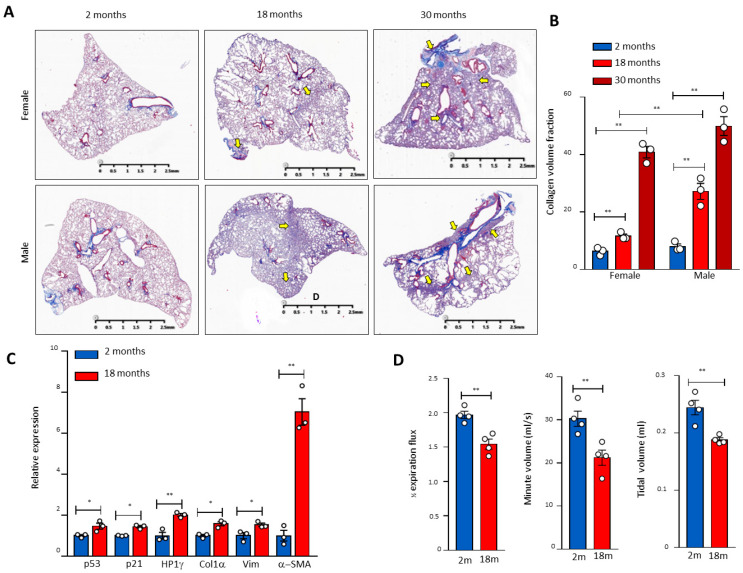
Age-related pulmonary fibrosis in mice with compromised lung respiratory function and activation of the p53 pathway. (**A**) Masson’s staining of mouse pulmonary sections of different genders and ages. Arrows indicate pulmonary fibrosis. (**B**) Quantitation of the pulmonary fibrosis by ImageJ. Three mice per group. (**C**) RNA was extracted from 2- and 18-month-old lung tissues, and the gene expression of p21, p53, HP1γ, collagen (Col1α), Vimentin (Vim), and α-SMA was determined by real-time PCR and graphed as fold-activation value based on three mice in each group. (**D**) The mouse respiratory functional analysis of different ages. The 1/2 expiration flux, tidal volume, and minute volume were determined by the eSpira Forced Manoeuvers System. Four mice per group. * and ** indicate statistical significance *p* < 0.05 and *p* < 0.01 respectively.

To investigate the molecular mechanisms of telomere DDR and SALI in pulmonary ageing and IPF, we examined the p53 signalling pathway in naturally aged mouse lung tissues and found that pulmonary fibrosis occurred with the increase in age in male mice (Figure 1A). In male mice aged 30 months, the pulmonary morphology confirmed the IPF-like fibrosis with significantly reduced volumes (Figure 1A,B). In association with pulmonary fibrotic lesions including alveolar fusion (Figure 1A), the mRNA levels of p53, p21, heterochromatin protein 1γ (HP1γ), type I collagen (Col1α), Vimentin (Vim), and α-SMA were increased significantly in the lung tissues of 18-month-old mice compared with 2-month-old mice (Figure 1C). Significantly compromised pulmonary respiratory functions were confirmed with impaired expiratory influxes and tidal and minute ventilation volumes in mice aged 30 months (Figure 1D). These results indicate that naturally aged mice develop pulmonary senescence with DDR and activation of the p53-related signalling pathway.

### 3.2. The Third Generation of Telomerase RNA Subunit Deficiency Causes Pulmonary Senescence and Low-Grade Inflammation

**Figure 2:**
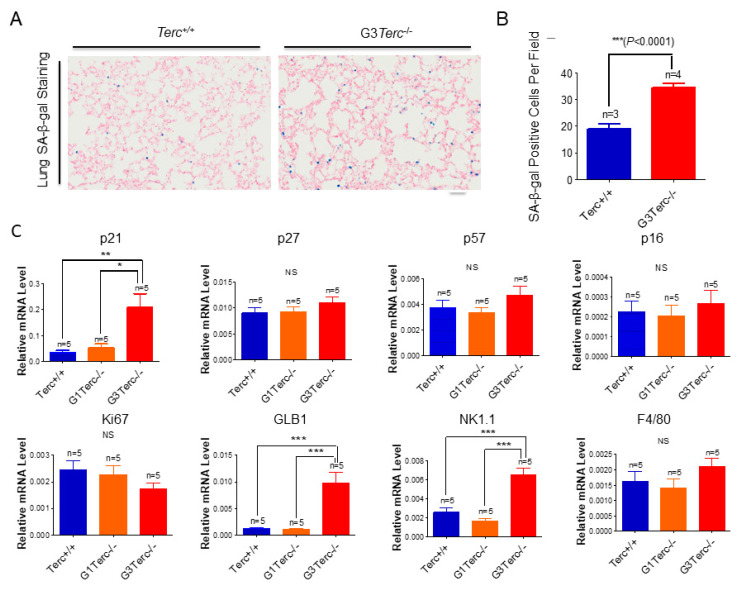

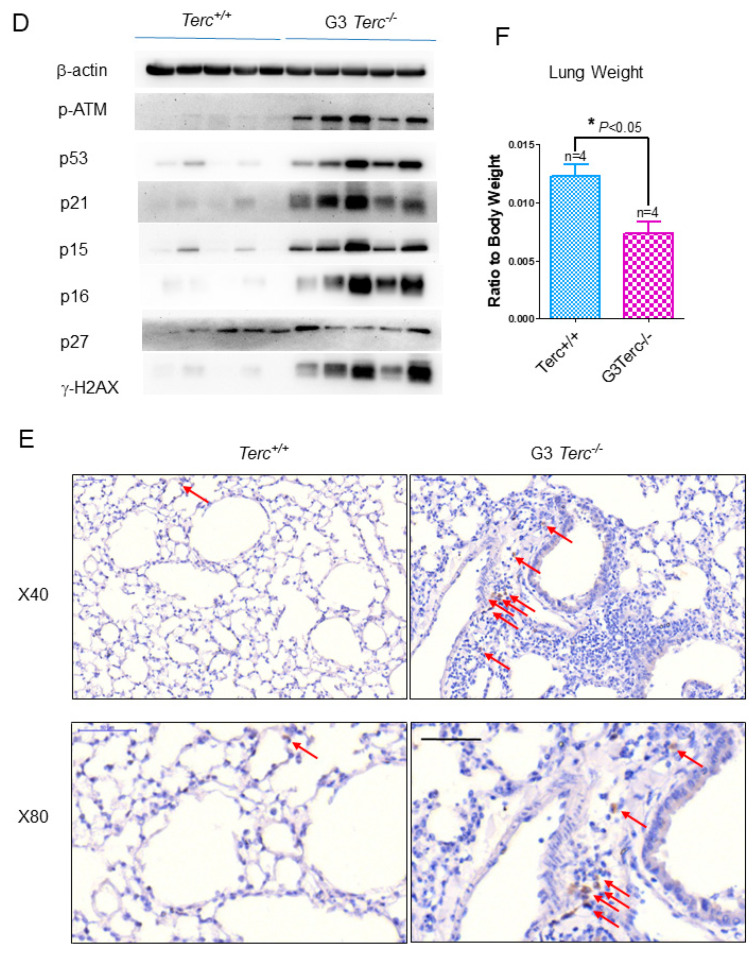
Significant increases in SA-β-gal staining, expression of cell cycle inhibitor p21, lysosomal β-galactosidase-associated GLB1, and natural killer cell marker NK1.1 in G3*Terc*^−/−^ mouse lungs. (**A**) Representative SA-β-gal-stained lungs of *Terc^+/+^* and G3*Terc*^−/−^ (magnification: 20×). Scale bar = 100 µm. (**B**) Quantification of SA-β-gal-stained cells by cell counting (as in A). Data are mean ± SE values based on the indicated number of lungs. (**C**) qPCR analysis of the indicated genes with RNA isolated from the indicated number of lungs by determining the relative mRNA level (ratio to β-actin). (**D**) Western blotting analysis with proteins from the lung tissues. (**E**) Representative NK1.1-stained lungs of *Terc^+/+^*, G3*Terc*^−/−^ (magnification: 40× and 80×). Scale bar = 50 µm. (**F**) The analysis of lung weight change by calculating the ratio to body weight based on the indicated number of mice. Statistical significance is indicated by * *p* < 0.05, ** *p* < 0.01, *** *p* < 0.001.

To determine the overall molecular signatures of pulmonary ageing in lung sections of telomerase-deficient mice (Figure 2A–E, Supplementary Figures S1 and S2), we found that, in addition to increased senescence marker SA-β-gal-positive cells in the G3 *Terc* knockout (KO) lungs compared with the wild-type (WT) littermate lungs, the mRNA levels for the cell cycle inhibitor p21 were markedly increased (Figure 2A–C). While no significant mRNA change was observed for p27, p57, p16, or cell proliferation index Ki67, potentially due to sample preparation in this instance, a marked protein increase was detected for phospho-ATM (p-ATM), p53, p21, p16, p15, and γH2AX (Figure 2C,D) in line with our previous observations at the mRNA levels [20]. The lysosomal β-galactosidase-associated GLB1 was increased in line with increased SA-β-gal (Figure 2C), and a marked increase in p21 was confirmed by Western blot (Figure 2D), suggesting that p21 might mediate telomere shortening-induced pulmonary senescence. In line with pulmonary senescence, the lung weights were significantly decreased (Figure 2F). In addition, we found that the natural-killer cell marker NK1.1 was significantly increased, whereas the macrophage marker F4/80 was not changed significantly (Figure 2C,E), suggesting that telomere shortening results in the activation of innate immunity with increased natural killer cells rather than macrophages. 

### 3.3. Pulmonary AEC2 Stem Cells Undergo Senescence by Mechanisms Involving p21 in Mice Deficient of the Telomerase RNA Component

**Figure 3:**
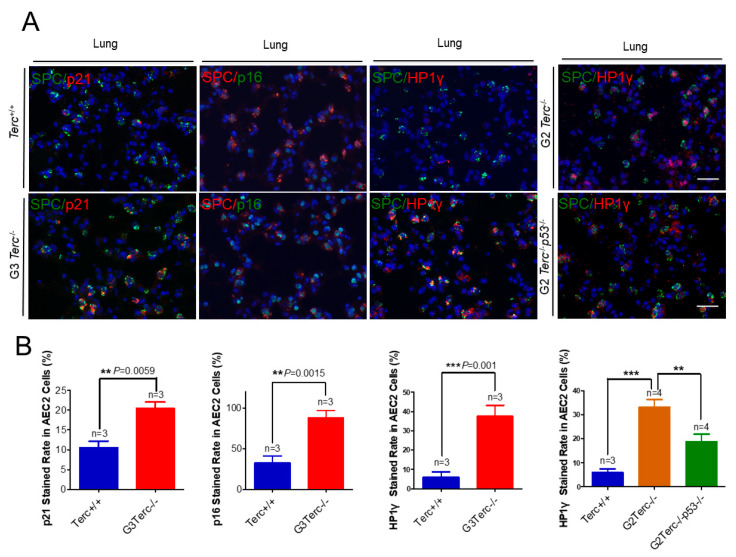
Marked increases in p21, p16, and SAHF-associated Hp1γ staining rates in AEC2 cells from late-generation *Terc*^−/−^ lungs, and p53 deletion significantly reversed increased Hp1γ expression. (**A**) Representative double immunofluorescence staining of *Terc^+/+^*, G3*Terc*^−/−^, G2*Terc*^−/−^, and G2*Terc*^−/−^*p53*^−/−^ mouse lungs (magnification: 40×) for SPC along with p21, p16, or Hp1γ. Scale bar = 30 µm. (**B**) Quantification of p21-, p16-, and Hp1γ-stained AEC2 cells by cell counting (as in A). Data are mean ± SE values based on the indicated number of lungs. Statistical significance is indicated by ** *p* < 0.01, *** *p* < 0.001.

To identify the cell type(s) showing a senescent phenotype, we performed double immunofluorescence staining on pulmonary alveolar stem AEC2 cells by SPC (surfactant protein C) and each of p21, p16, or Hp1γ. We found that the cell cycle inhibitor p21 was significantly higher in AEC2 cells of the lung sections in G3 *TERC* KO mice versus the WT (20.44% vs. 10.54%, respectively) (Figure 3). In addition, staining with the cell cycle inhibitor p16 in AEC2 cells showed that p16 was significantly higher in AEC2 cells of G3 *TERC* KO lungs (88%) compared with the WT (32.7%) (Figure 3). Consistent with the increased cell cycle inhibitors in AEC2 cells, senescence-associated heterochromatin foci (SAHF)-associated HP1γ were increased. We found that HP1γ was significantly higher in the lungs of G3 *TERC* KO than that in the WT (37.5% vs. 6.0%) (Figure 3). The telomere lengths of AEC2 stem cells were significantly shortened in late-generation *TERC* KO lungs [20], suggesting that telomere shortening triggers AEC2 cell cycle arrest and senescence by mechanisms involving p21 in mouse lungs.

### 3.4. Marked Increase in Apoptosis-Related Cleaved Caspase-3 Staining in AEC2 Cells from G3 TERC Knockout Mouse Lungs

**Figure 4:**
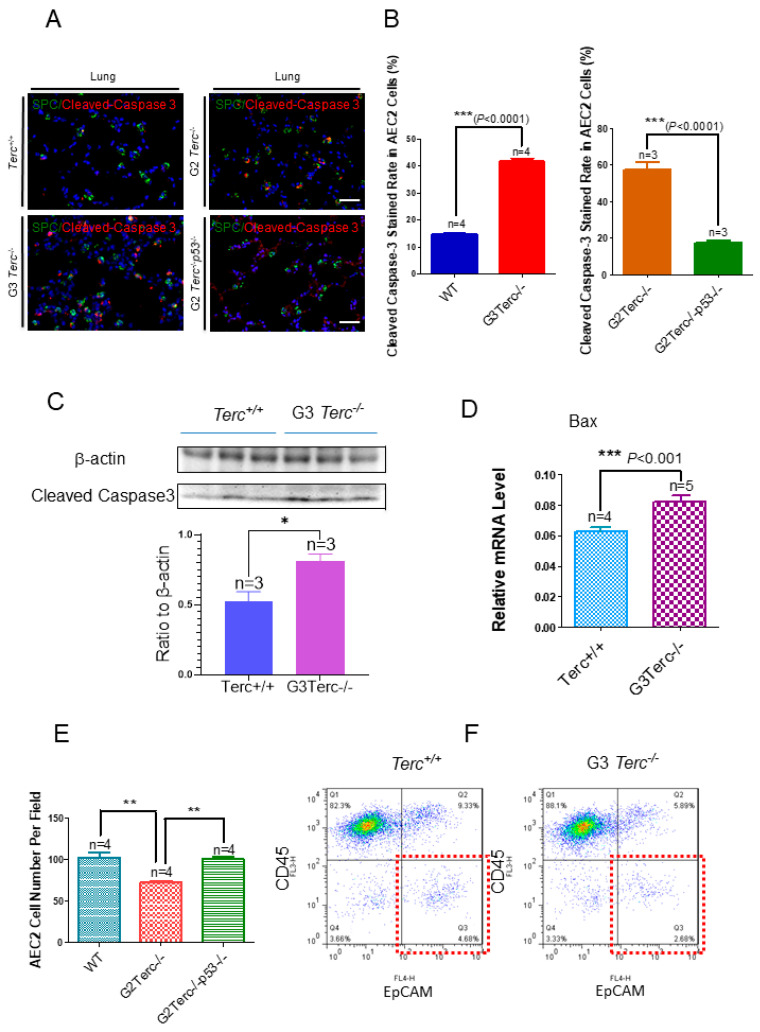
Marked increases in apoptosis-related cleaved caspase-3 staining rates in the AEC2 cells from late-generation *Terc*^−/−^ mouse lungs and rescued in *p53*^−/−^. (**A**). Representative double immunofluorescence staining of WT, G3*Terc*^−/−^, G2*Terc*^−/−^, and G2*Terc*^−/−^*p53*^−/−^ mouse lungs (magnification: 40×) for SPC and cleaved caspase-3. Scale bar = 30 µm. (**B**). Quantification of cleaved caspase-3 stained AEC2 cells by cell counting (as in (**A**)). Data are mean ± SE values based on the indicated number of lungs. (**C**). Marked increases in apoptosis with cleaved caspase-3 in the late-generation *Terc*^−/−^ mouse lungs by Western blotting analysis. (**D**). Increases in apoptosis-related Bax mRNA levels in the late-generation *Terc*^−/−^ mouse lungs by RT-qPCR analysis. Data are mean ± SE values based on the indicated number of lungs. (**E**). Significant decrease in the AEC2 cell number by cell counting in the slides from late-generation *Terc*^−/−^ mouse lungs, and rescued in *p53*^−/−^. Data are mean ± SE values based on the indicated number of lungs. (**F**). Representative flow cytometry result showing significant decreases in AEC2 cell number (gated by CD45^−^EpCAM^+^) in late-generation *Terc*^−/−^ mouse lungs. Statistical significance is indicated by * *p* < 0.05, ** *p* < 0.01, *** *p* < 0.001.

To investigate the effect of TERC deficiency on apoptosis in AEC2 cells in the lungs, double immunofluorescence staining was performed for the AEC2-specific marker, SPC, and the apoptotic marker, cleaved caspase-3. Staining of cleaved caspase-3 in AEC2 cells showed significantly increased levels in the lungs of G3 *TERC* KO mice compared with that in WT lungs (41.8% vs. 14.8%, respectively) (Figure 4A,B). In addition, the levels of cleaved caspase-3 protein and apoptosis-related Bax [31,32] were increased significantly in the lungs of G3 *TERC* KO mice (Figure 4C,D). The AEC2 cell number was decreased significantly in the lungs of G3 *TERC* KO mice by cell counting in slides (SPC-positive cells) or flow cytometry (Figure 4E,F). These results suggest that TERC deficiency triggers not only alveolar stem cell senescence but also apoptosis by a mechanism involving p21 and p16 in lungs. 

### 3.5. AEC1 Cell-Associated T1α Is Significantly Elevated in Both G3 TERC Knockout Mouse Lungs and AEC2 Cells 

**Figure 5:**
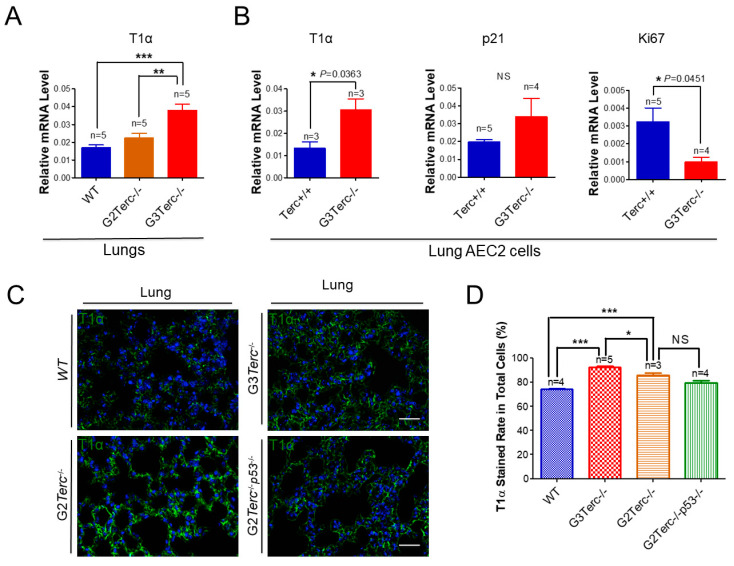
Significant elevation of AEC1 cell marker T1α in both late-generation mouse lungs and isolated AEC2 cells, which was not rescued by *p53*^−/−^. (**A**) qPCR analysis of T1α mRNA levels from the indicated number of lungs. Data are expressed as mean ± SE values. (**B**) qPCR analysis of T1α mRNA levels with AEC2 cell RNAs isolated from lungs by FACS sorting. Data are expressed as mean ± SE values based on the indicated animal numbers used to isolate lung AEC2 cells. (**C**) Representative T1α immunofluorescence staining of WT, G3*Terc*^−/−^, G2*Terc*^−/−^, and G2*Terc*^−/−^*p53*^−/−^ mouse lungs (magnification: 40×). Scale bar = 30 µm. (**D**) Quantification of T1α stained rate by cell counting (as in (**A**)). Data are mean ± SE values based on the indicated number of lungs. Statistical significance is indicated by * *p* < 0.05, ** *p* < 0.01, *** *p* < 0.001.

As an AEC1 canonical marker in the lungs [33], T1α mRNA levels were up-regulated more than two-fold in the lungs of G3 *TERC* KO mice in comparison with that of WT animals (Figure 5A). Consistent with an effect of telomere shortening, the T1α mRNA level was significantly higher in the lungs of G3 *TERC* KO mice than that of G2 *TERC* KO mice (Figure 5A). In addition, T1α was increased in the AEC2 cells of G3 *TERC* KO mice compared with the WT (Figure 5A,B), suggesting that telomere shortening promotes differentiation from AEC2 cells to AEC1 cells while triggering AEC2 cell senescence and apoptosis. Consistently, p21 mRNA was up-regulated whereas Ki67 [34,35] was down-regulated in the lungs of G3 *TERC* KO compared with the WT (Figure 5B). Moreover, the mRNA level of another AEC1 marker, aquaporin 5 (AQP5), was significantly increased in the lungs of G3 *TERC* KO mice compared with that of WT mice (Supplementary Figure S2). These results suggest that AEC2 cells undergo differentiation in G3 *TERC* KO mice.

### 3.6. Rescue of Alveolar Stem Cell Senescence and Apoptosis in the Late Generation of TERC KO Mice by p53 Deficiency

To understand the molecular mechanism of AEC2 cellular senescence and apoptosis caused by telomere shortening, mice carrying the *Terc^−/−^* allele were crossed with the *p53^−/−^* allele to generate double-knockout mice of *TERC* and *p53*. A comparison of the SAHF-associated Hp1γ expression in AEC2 cells of G2 *TERC* and *p53* double-knockout mouse lungs with that of G2 *TERC* KO mouse lungs by immunofluorescence double staining showed that the increased Hp1γ staining in AEC2 cells in G2 *TERC* KO mouse lungs compared with that of WT (33.2% vs. 6.03%) was significantly decreased by p53 knockout in AEC2 cells (18.80%) (Figure 3A,B), suggesting that p53 mediated AEC2 senescence induced by TERC deficiency. In addition, staining of cleaved caspase-3 in AEC2 cells showed that increased cleaved caspase-3 in G2 *TERC* KO AEC2 was significantly decreased in G2 *TERC* and *p53* double-knockout AEC2 (57.1% vs. 17.1%) (Figure 4A,B), suggesting that, in addition to cell senescence, AEC2 apoptosis induced by TERC deficiency is mediated by a p53-dependent mechanism. Furthermore, staining for the AEC1 cell marker T1α showed that increased T1α in G2 *TERC* KO AEC2 cells was not affected by G2 *TERC* and *p53* double knockouts in the mouse lungs (Figure 5C,D), suggesting that TERC KO-induced T1α elevation in AEC2 is mediated by a mechanism independent of p53.

## 4. Discussion

Telomeres are nucleotide–protein complexes that maintain stable chromosome ends [36]. In mammalian somatic cells, telomere length becomes progressively shorter with each cell division due to the DNA end replication problem, but telomere length is maintained by telomerase activity [37]. In order to study the impacts of telomere shortening, several telomerase knockout mouse models have been generated, and late generations of *TERC* knockout mice possess significantly shorter telomeres [19]. The abnormalities identified in lung tissues in the *TERC* knockout model include poor alveolar integrity, compromised regenerative ability under partial pneumonectomy, and pulmonary fibrosis induced by telomere dysfunction and shortening [20,24,38,39]. In the present study, we demonstrate that naturally aged mice develop pulmonary senescence and IPF-like lesions with activation of the p53 signalling pathway. The DNA damage response is activation by telomerase deficiency in the AEC2 stem cells as a major population of cellular senescence with increased staining of the cell cycle inhibitors p21, p16, and SAHF-associated Hp1γ in the lungs of mice after three generations of telomerase RNA component deficiency. The data confirmed previous findings that telomere shortening promotes pulmonary stem cell senescence, consistent with a common cellular basis for the development of telomere shortening-associated diseases in the respiratory system such as IPF and COPD [20,24]. In addition, we demonstrate that TERC deficiency also triggers AEC2 cell apoptosis with increased apoptosis-associated cleaved caspase-3, suggesting that the reduced population of AEC2 stem cells in telomerase deficiency [20] is due to not only permanent arrest of the cell cycle but also increased apoptotic cell death. Moreover, this study reveals that both AEC2 cell senescence and AEC2 cell apoptosis are dependent on p53, which is consistent with previous observations supporting a role for p53 as a central control mechanism for both cell senescence and apoptosis that is promoted by defects in telomeres [40] as well as mitochondrial dysfunction [41]. 

At the whole organ level of late-generation *TERC* KO mouse lungs, besides high levels of cellular senescence features including increased p21 and SA-β-gal activities in association with GLB1, the natural killer cell marker NK1.1 is strongly up-regulated in G3 *TERC*-deficient lungs. Consistent with the involvement of innate immune cells, especially natural killer cells, our previous studies demonstrated significant cytokine storms in the lungs of telomerase deficiency, including markedly elevated IL-1, IL-6, CXCL15, IL-10, TNF-alpha, and CCL2, with IL-6 and CXCL15 spillover into the bronchial alveolar lavage fluids [20]. An increased infiltration of NK cells may thus provide a clue in further investigations into telomerase deficiency-associated SALI for potential intervention strategies. It is possible that compromised telomere maintenance triggers epigenetic alterations, resulting in altered gene expressions and DNA damage-related immune response serving as DNA damage-associated molecular patterns in SALI and lung ageing [26,42].

Previous studies demonstrated that telomere maintenance plays a significant role in regulating fibroblast transdifferentiation in fibrotic pathogenesis [27]. In an attempt to determine the effect of TERC deficiency on AEC2 differentiation, we demonstrate that the AEC1 marker T1α levels are surprisingly increased in the lungs of the late-generation *TERC* knockout mice and in isolated AEC2 cells. Since T1α is an apical membrane protein required in AEC1 cells as a differentiation marker for AEC1 cells [3,33], our data suggest that telomere shortening promotes cellular differentiation from AEC2 cells to AEC1 cells. Moreover, these results show that T1α up-regulation is not mediated by p53 as p53 deletion does not inhibit T1α. It is possible that telomere shortening leads to altered gene expressions via telomere position effect [43] and/or alternative splicing of a suite of genes related to genome stability, DNA repair, and chromosome remodelling [44]. Thus, TERC deficiency-caused telomere maintenance failure may result in a heterogeneous population of AEC2 with not only increased senescence and apoptosis but also enhanced differentiation. Further studies are required to determine the mechanisms controlling AEC2 fate decisions, e.g., between senescence and apoptosis; between differentiation and transdifferentiation; between senescent cell resistance and susceptibility to cell death; and between different forms of cell deaths such as apoptosis, pyroptosis, and autophagic cell death [45,46]. Studies are also required to determine if an alternative anti-apoptotic function of human TERC involves hTERP encoded by *TERC*, as reported recently [21].

In conclusion, pulmonary ageing and IPF-like lesions occur in the natural ageing process whereby pulmonary stem cells mediate telomerase deficiency-induced SALI, causing pulmonary senescence and fibrosis. Cellular mechanisms of pulmonary senescence include increased senescence, apoptosis, and differentiation of the alveolar stem cells. AEC2 stem cell senescence and apoptosis are mediated at least in part by the tumour suppressor protein p53-dependent mechanisms involving p21, whereas AEC2 differentiation is p53-independent, providing a molecular basis for further investigation in age-related lung diseases such as IPF and COPD. 

## Data Availability

Not applicable.

## Supplementary Materials

The following are available online at https://www.mdpi.com/article/10.3390/cells10112892/s1, Figure S1: RT-qPCR analysis of *Terc* and *Tert* mRNA levels in late-generation *Terc*^−/−^ mouse lungs, Figure S2: Significant increased mRNA levels of AEC1 marker AQP5 in late-generation *Terc*^−/−^ mouse lungs, Table S1: Primer sequences used for real-time qPCR analysis.
