# Supplementary material for: Pulmonary Alveolar Stem Cell Senescence, Apoptosis, and Differentiation by p53-Dependent and -Independent Mechanisms in Telomerase-Deficient Mice
Source: Cells. 2021 Oct 26;10(11):2892. doi: 10.3390/cells10112892 (PMC8616483; doi:10.3390/cells10112892)
Supplement: Supplementary file 1 [file cells-10-02892-s001.zip › cells-1383402 Suppl figures and table/cells-1383402 Suppl Table S1 1025.pdf]

Suppl. Table S1. Primer sequences used for real-time qPCR analysis

**Gene**	Oligo Name	**Primer sequences**
P21	q-m-p21-f	forward, 5'-CCAATCCTGGTGATGTCCG-3'
	q-m-p21-r	reverse, 5'-TCAAAGTTCCACCGTTCTCG-3'
P27	q-m-p27-f	forward, 5'-GTGGACCAAATGCCTGACTC-3'
	q-m-p27-r	reverse, 5'-TCTGTTCTGTTGGCCCTTTT-3'
P53	q-m-p53-f	forward, 5'-CCCCTGTCATCTTTTGTCCCT-3'
	q-m-p53-f	reverse, 5'-AGCTGGCAGAATAGCTTATTGAG-3'
P57	q-m-p57-f	forward,5'-GGAGCAGGACGAGAATCAAG-3'
	q-m-p57-r	reverse,5'-GTTCTCCTGCGCAGTTCTCT-3'
P16	q-m-p16-f	forward, 5'-CATCTGGAGCAGCATGGAGTC-3'
	q-m-p16-r	reverse,5'-GGGTACGACCGAAAGAGTTCG-3'
Ki67	q-m-Ki67-f	forward, 5'-GCTGTCCTCAAGACAATCATCA-3'
	q-m-Ki67-r	reverse, 5'-GGCGTTATCCCAGGAGACT-3'
GLB1	q-m-Glb1-f	forward, 5'- ATACATCTCGGGAAGCATTCAT-3'
	q-m-Glb1-r	reverse, 5'- GGTCCCCAGAAAACTCATATTG-3'
NK1.1	q-m-NK1.1-f	forward, 5'-GCTGTGCTGGGCTCATCCT-3'
	q-m-NK1.1-r	reverse, 5'-TTGATGGTTTTTGTACTAAGACTCGCA-3'
F4/80	q-m-F4/80-f	forward, 5'-GATACAGCAATGCCAAGCAGT-3'
	q-m-F4/80-f	reverse, 5'-TTGTGAAGGTAGCATTCACAAGTGTA-3'
Podoplanin (T1α)	q-m-T1 α -f	forward, 5'-TCCACCTCAGCAACCTC-3'
	q-m-T1 α -r	reverse, 5'-GCTAACAAGACGCCAACTA-3'
AQP5	q-m-AQP5-f	forward, 5'- CTCCGAGCCATCTTCTACGT-3'
	q-m-AQP5-r	reverse, 5'- CCTGGTGTTGTGTTGTTGCT-3'
Bax	q-m-Bax-f	forward, 5'-TGTTTGCTGATGGCAACTTC—3'
	q-m-Bax-r	reverse, 5'-GATCAGCTCGGGCACTTTAG-3'
Terc	q-m-Terc-f	forward, 5'-TCATTAGCTGTGGGTTCTGGT-3'
	q-m-Terc-r	reverse, 5'-TGGAGCTCCTGCGCTGACGTT-3'
Tert	q-m-Tert-f	forward, 5'-GGATTGCCACTGGCTCCG-3'
	q-m-Tert-r	reverse, 5'-TGCCTGACCTCCTCTTGTGAC-3'
HP1γ	q-m-HP1γ-f	forward, 5'-ACTGGACCGTCGTGTAGTGAA-3'

	q-m-HP1γ-r	reverse, 5’-GCCCCTTGGTTTGTCAGCA-3’
Col1α	q-m-Col1α-f	forward, 5’-GCTCCTCTTAGGGGCCACT-3’
	q-m- Col1α-r	reverse, 5’-ATTGGGGACCCTTAGGCCAT-3’
Vimentin	q-m-Vimentin-f	forward, 5’-CGGCTGCGAGAGAAATTGC-3’
	q-m-Vimentin-r	reverse, 5’-CCACTTTCCGTTCAAGGTCAAG-3’
α-SMA	q-m-α-SMA-f	forward, 5’-ATTGTGCTGGACTCTGGAGATGGT-3’
	q-m-α-SMA-r	reverse, 5’-TGATGTCACGGACAATCTCACGCT-3’
Beta-actin	q-m-beta-actin	forward, 5’- CAGCCTTCCTTCTTGGGTAT -3’
	q-m-beta-actin	reverse, 5’- TGGCATAGAGGTCTTTACGG -3’
