# Supplementary figures and images for: Pulmonary Alveolar Stem Cell Senescence, Apoptosis, and Differentiation by p53-Dependent and -Independent Mechanisms in Telomerase-Deficient Mice
Source: Cells. 2021 Oct 26;10(11):2892. doi: 10.3390/cells10112892 (PMC8616483; doi:10.3390/cells10112892)

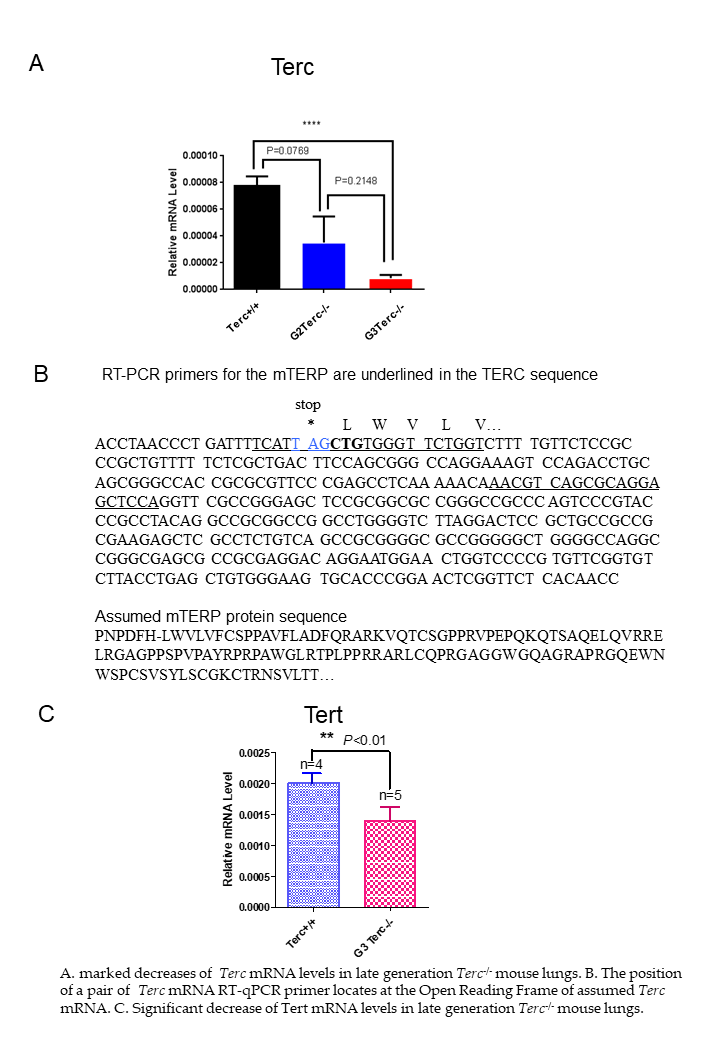

Supplement: Supplementary file 1 [file cells-10-02892-s001.zip › cells-1383402 Suppl figures and table/cells-1383402 Suppl Figure S1 1025.tif]

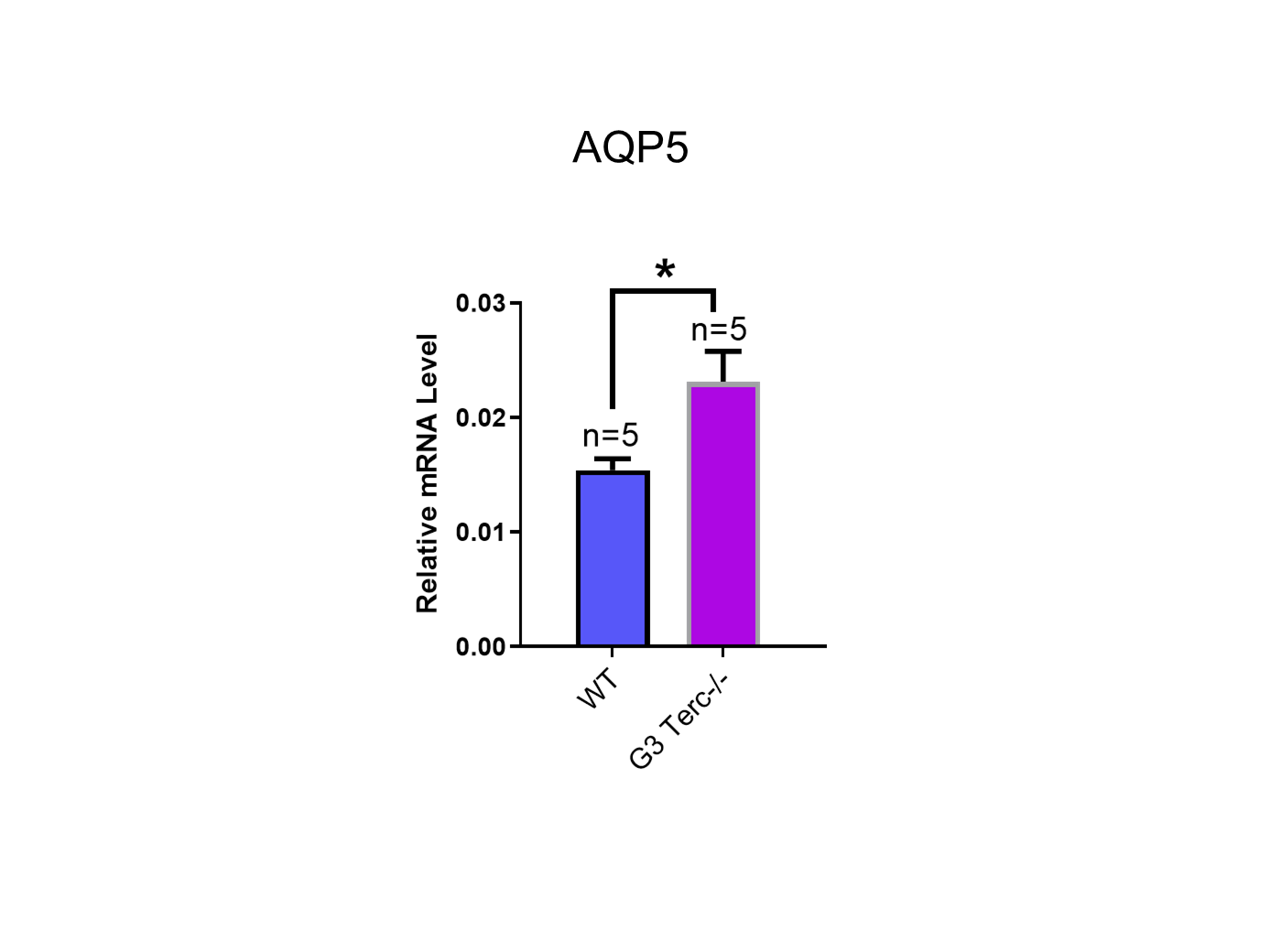

Supplement: Supplementary file 1 [file cells-10-02892-s001.zip › cells-1383402 Suppl figures and table/cells-1383402 Suppl Figure S2 1025.tif]
